# Outcomes of Non-operative Management of Neck of Femur Fracture: A Single Center Study

**DOI:** 10.7759/cureus.91096

**Published:** 2025-08-27

**Authors:** Tithi Jain, Rajesh Bawale, Rory McCabe, Giles Faria, Bijayendra Singh

**Affiliations:** 1 Trauma and Orthopaedics, Medway Maritime Hospital, Medway NHS Foundation Trust, Gillingham, GBR; 2 Trauma and Orthopaedics, East Kent Hospitals University NHS Foundation Trust, Canterbury, GBR

**Keywords:** fracture, management, neck of femur, ortho-surgery, outcome

## Abstract

Objectives

This study aimed to understand the incidence, demographics, inpatient stay duration, role of imaging and outcomes for patients diagnosed with neck of femur (NOF) fractures and managed non-operatively.

Methods

In this study, the data was collected retrospectively for 14 years (January 2009-January 2023). The inclusion criteria involved all non-operatively managed NOF fractures. The trauma board, electronic patient records, radiographs, and National Hip Fracture Database (NHFD) were used for the data collection. The data was collected as demographic details, fracture classification, any reasons for non-operative management, mortality and further surgical management. Patients who had surgery, died or transferred to other sites for specialist surgery were excluded.

Results

In our study, 1.5% (62/4132) of NOF fractures were managed non-operatively at our institution. The reasons for non-operative management were as follows: medically unwell patients (45.2%, n = 28) in whom operative risk was thought to outweigh benefit and risk of death was high within the 48-72 hrs of admission. The second group of patients had minimal or no pain and had old fractures with comfortable mobilisation (54.8%, n = 34). Out of 34/62 patients who were mobilised, 14.7% (5/34) of this patient subgroup subsequently required surgical intervention for failed non-operative management. In the medically unwell group (28/62), the 30-day mortality was 42.8% (12/28) with a 1-year mortality rate of 60.7% (17/28). The patients who were mobilised (34/62), the 30-day mortality was 11.8% (4/34), with a 1-year mortality rate of 14.7% (5/34). The combined average 1-year mortality for this cohort was 35.4% (22/62).

Conclusions

Our study showed a higher mortality rate for the medically unwell group (42%). Among patients with stable fractures that allowed comfortable mobilisation, the 1-year mortality rate was 20%. Additionally, 14% of these patients (5 out of 34) eventually required surgical intervention. We note that an analysis is necessary to assess the functional outcomes of this subgroup, as well as the potential cost implications. The combined average 1-year mortality for this cohort was 35% (22/62), which was due to the high-risk factors in the medically unwell group.

## Introduction

Fracture of the femoral neck is a common and serious injury, particularly among the elderly population. It is associated with significant morbidity, mortality, and socioeconomic burden [1]. The incidence of hip fractures is increasing globally due to the aging population, with an estimated 6.26 million cases expected annually by 2050 [2].

Traditionally, operative management has been the standard of care for femoral neck fractures, with the primary goals of early mobilization, pain relief, and restoration of function [3]. Common surgical interventions include internal fixation, hemiarthroplasty, or total hip arthroplasty, depending on patient factors and fracture characteristics [4].

However, there exists a subset of patients for whom operative management may not be feasible or appropriate. These include individuals with severe comorbidities, limited life expectancy, non-ambulatory status prior to injury, or those who decline surgery [5]. In such cases, non-operative management becomes a consideration, although it is generally associated with higher rates of complications and mortality [6].

Non-operative management typically involves pain control, bed rest, and gradual mobilization as tolerated [7]. While this approach avoids the risks associated with surgery and anesthesia, it can lead to prolonged immobilization, potentially resulting in complications such as pressure sores, pneumonia, and venous thromboembolism [8].

Despite the importance of understanding the outcomes of non-operative management in this patient population, there is a paucity of recent literature focusing on this approach. Most studies have concentrated on surgical outcomes, leaving a gap in our knowledge regarding the natural history and prognosis of conservatively managed femoral neck fractures [9].

This single-centre study aims to evaluate the outcomes of non-operative management in patients with femoral neck fractures. By examining factors such as mortality rates, functional status, pain levels, and complications, we hope to provide valuable insights that can inform decision-making and improve patient care in cases where surgery is not a viable option.

## Materials and methods

This study included patients admitted and treated at a level 2 trauma center between January 2009 and January 2023. Over this 14-year period, we identified a total of 62 patients who sustained femoral neck fractures and were managed non-operatively. These patients formed the basis of our investigation.

To be included in the study, patients needed to meet specific criteria. They had to be aged 60 years or older and have a femoral neck fracture confirmed through radiological imaging. Additionally, the patients must have been managed non-operatively - either due to their own preference, medical reasons that contraindicated surgery, or other considerations - and they needed to have been followed for at least 12 months after injury or until death, whichever came first.

Certain patients were excluded from the study to maintain clarity and consistency of our results. Those with pathological fractures (fractures caused by disease rather than trauma) were not included. Patients who initially began non-operative management but then underwent surgery within the first month were also excluded, as were those whose medical records were incomplete or insufficient for analysis.

We carefully reviewed medical records to gather detailed information about each patient. This included demographic factors such as age, sex, and pre-injury activity levels. To better understand their overall health, we assessed comorbidities using the Charlson Comorbidity Index, which helps quantify patients’ existing medical conditions. We also examined fracture-specific details by reviewing radiographs, classifying the fractures according to the Garden classification system, and noting whether there was displacement. The treatment approach for each patient - whether operative or non-operative - was documented alongside the reasons behind the choice of non-operative management.

For patients managed without surgery, the care pathway focused on pain control through appropriate analgesia and initially encouraged rest. This was followed by gradual mobilization supported by physiotherapy as tolerated. To minimize the risk of blood clots, low molecular weight heparin was administered for deep vein thrombosis prophylaxis unless there were contraindications. Preventative measures against pressure ulcers were also rigorously applied throughout the patient’s care.

Patients were monitored regularly after their injury. Follow-up appointments were conducted in outpatient clinics or via telephone for those unable to attend in person. Our primary outcome measure was survival, assessed at 30 days and again at 1 year post-injury. We also evaluated several secondary outcomes, including:

Functional status, measured by the Modified Barthel Index (MBI), which assesses independence in daily activities.

Pain levels, recorded using the Visual Analog Scale (VAS), a standard tool for quantifying subjective pain.

Incidence of complications such as pressure sores, pneumonia, urinary tract infections, and venous thromboembolism.

The rate of delayed surgery - defined as surgery performed more than one month after the initial injury.

Length of hospital stay

For those patients who survived and were able to attend follow-up visits, we also assessed radiographic healing. Union was defined as the presence of bridging callus on at least three cortices visible in orthogonal radiographic views, indicating that the fracture had successfully healed.

Statistical analysis

All collected data were analyzed using SPSS version 25.0 (IBM Corp., Armonk, NY, USA). Continuous variables, such as age and length of stay, were described using means and standard deviations. Categorical variables, like sex and complication rates, were summarized as frequencies and percentages. To analyze survival over time, we used the Kaplan-Meier method. Factors associated with mortality were further examined using Cox proportional hazards regression modeling. Changes in functional status and pain scores over multiple time points were investigated using repeated measures analysis, allowing us to track trends.

## Results

Among the 4,132 patients treated for femoral neck fractures at our hospital over a 14-year period, only 62 individuals (1.5%) received conservative (non-operative) treatment. This subgroup had a mean age of 84.3 years, and 67.7% (42 out of 62) were female.

These non-operatively managed cases fell into two main categories. Twenty-eight patients (45.2%) were deemed medically unfit for surgery due to poor overall health, and their treating physicians concluded that the risks of operative intervention outweighed the potential benefits. The remaining 34 patients (54.8%) sustained appendage fractures associated with minimal or no pain; some of these individuals were even able to ambulate independently, suggesting the presence of old, stable injuries.

Interestingly, a majority of these conservatively treated patients (59.6%) sustained Garden type III or IV fractures - classifications that are typically managed surgically. This deviation from standard practice is detailed in Table 1, which outlines the distribution of fracture types within the non-operative cohort.

**Table 1:** Classification of Patients (14-Year Review)

Classification	%
Medically unwell, non-operative management	28 (45.2%)
Minimal/no pain and old fractures with mobilization	34 (54.8%)
Garden Classification: Garden I or II	25 (40.3%)

The one-year mortality rate for the entire non-operatively managed group was 35.5% (22 out of 62 patients), reflecting the complex medical conditions frequently present in this population. Outcomes varied notably between the two subgroups. Patients deemed medically unfit for surgery had a significantly higher mortality rate, with 42.8% dying within 30 days and 60.7% within one year. In contrast, patients who remained ambulatory with minimal symptoms (the mobilized group) demonstrated substantially lower mortality rates - 11.8% at 30 days and 14.7% at one year. Additionally, 14.7% of patients in the mobilized group ultimately required surgical intervention due to failure of conservative treatment. These subgroup-specific outcomes are summarized in Table 2.

**Table 2:** Outcomes by Management Group

Outcome	Medically Unwell (n=28)	Mobilized Group (n=34)	P value	Test Statistic (χ²)
30-day mortality	12 (42.8%)	4 (11.8%)	0.005	7.75
1-year mortality	17 (60.7%)	5 (14.7%)	<0.001	14.26
Required subsequent surgery	N/A	5 (14.7%)	NA	NA

Medical complications in the non-operatively managed group are detailed in Table 3. Urinary tract infections were the most frequently reported condition (29.03%), followed by pneumonia (22.6%) and pressure ulcers (17.7%). These findings suggest that delayed mobilisation in this cohort may have contributed to the development of such complications, underscoring the potential risks associated with conservative management in elderly patients.

**Table 3:** Complications in Non-operatively Managed Patients

Complications	Frequency (%)
Pressure ulcers	11 (17.7%)
Pneumonia	14 (22.6%)
Urinary tract infections	18 (29.0%)

Details regarding the patients who required delayed surgical intervention following initial conservative management are presented in Table 4. A total of five patients eventually underwent surgery; three received cemented hip hemiarthroplasty, while the remaining two underwent total hip replacement. The average time from injury to surgery was 6.2 weeks, with the interval ranging from 4 to 10 weeks depending on individual clinical progression.

**Table 4:** Subsequent Surgical Management

Type of Surgery	Frequency (%)
Hemiarthroplasty	3 (60%)
Total hip arthroplasty	2 (40%)
Total	5 (100%)

## Discussion

This retrospective single-centre study focuses on the outcomes of patients diagnosed with femoral neck fractures who were managed non-operatively. Our results demonstrated significant mortality rates and potential complications associated with this treatment approach, especially in patients with multiple underlying medical conditions.

Compared to the published literature, our study showed a low non-operative management rate of 1.5%. Moulton et al. reported that about 5% of patients with hip fractures received non-operative treatment, reflecting differences that may arise from the diverse patient populations served by each hospital and the variations in healthcare systems [10].

Our study revealed a clear difference in mortality rates between patients who were medically unstable and those who were able to mobilize early. The medically unwell group experienced 30-day and 1-year mortality rates of 42.8% and 60.7%, respectively, whereas the medically stable, early mobilization group had significantly lower mortality rates of 11.8% and 14.7%. These findings align with those reported by Moulton et al. [10], Gregory et al. [11], and Dedovic et al. [12], who documented similar mortality rates in non-operatively managed femoral neck fractures. Furthermore, Hansson et al. [13] conducted a prospective study of 81 elderly patients with displaced femoral neck fractures treated non-operatively, reporting similarly high mortality and poor functional outcomes, which provides important historical context and reinforces the fragility of this patient group.

Our results also showed that 14.7% of patients initially managed non-operatively in the mobilized group eventually required surgery due to failure of conservative treatment. This is comparable to the 18% delayed surgery rate reported by Pradhan et al. [14]. Differences in patient selection and follow-up duration might explain this variation.

In terms of complications, our observed rates of pressure ulcers (17.7%), pneumonia (22.6%), and urinary tract infections (29.03%) are consistent with previous literature addressing immobility-related risks in this population [8]. These findings emphasize the critical importance of careful nursing care and, where possible, early mobilization to minimize adverse outcomes.

A more recent study by Omura et al. [15] evaluated non-operative management outcomes in elderly patients with poor surgical candidacy, reporting similar challenges related to increased complications and mortality. Their findings highlight the necessity for vigilant monitoring and tailored care plans, which parallels our conclusions on the importance of close follow-up and timely surgical intervention when conservative management fails.

We noted that the average time to consider surgery after initial non-operative management failure was 6.2 weeks. Prior research by Haleem et al. [16] has linked delayed surgery in such cases to increased complication rates and worse functional recovery, underscoring the need for early decision-making in these patients.

Finally, we acknowledge the limitations of our study, including its retrospective design and single-centre setting, which may affect the generalizability of the findings.

## Conclusions

Our study concludes that non-operative management for femoral neck fractures is associated with high mortality rates and complications, especially in patients who are medically ill. However, it may be appropriate for a small subset of patients. To maximise results, careful patient selection, vigilant observation, and prompt intervention in instances of unsuccessful non-operative therapy are essential.

## References

[1] Veronese N, Maggi S (2018). Epidemiology and social costs of hip fracture. Injury.

[2] Gullberg B, Johnell O, Kanis JA (1997). World-wide projections for hip fracture. Osteoporos Int.

[3] Bhandari M, Swiontkowski M (2017). Management of acute hip fracture. N Engl J Med.

[4] Florschutz AV, Langford JR, Haidukewych GJ, Koval KJ (2015). Femoral neck fractures: current management. J Orthop Trauma.

[5] Jain R, Basinski A, Kreder HJ (2003). Nonoperative treatment of hip fractures. Int Orthop.

[6] Xu DF, Bi FG, Ma CY, Wen ZF, Cai XZ (2017). A systematic review of undisplaced femoral neck fracture treatments for patients over 65 years of age, with a focus on union rates and avascular necrosis. J Orthop Surg Res.

[7] Bukata SV, DiGiovanni BF, Friedman SM (2011). A guide to improving the care of patients with fragility fractures. Geriatr Orthop Surg Rehabil.

[8] Beaupre LA, Jones CA, Saunders LD, Johnston DW, Buckingham J, Majumdar SR (2005). Best practices for elderly hip fracture patients. A systematic overview of the evidence. J Gen Intern Med.

[9] Damany DS, Parker MJ, Chojnowski A (2005). Complications after intracapsular hip fractures in young adults. A meta-analysis of 18 published studies involving 564 fractures. Injury.

[10] Moulton LS, Green NL, Sudahar T, Makwana NK, Whittaker JP (2015). Outcome after conservatively managed intracapsular fractures of the femoral neck. Ann R Coll Surg Engl.

[11] Gregory JJ, Kostakopoulou K, Cool WP, Ford DJ (2010). One-year outcome for elderly patients with displaced intracapsular fractures of the femoral neck managed non-operatively. Injury.

[12] Dedovic Z, Talic-Tanovic A, Resic H, Vavra-Hadziahmetovic N (2013). Mortality among third age patients with hip fracture and high cardiac risk. Med Arh.

[13] Hansson S, Strömqvist B, Karlsson J, Hasserius R (1995). Nonoperative treatment of displaced femoral neck fractures in elderly patients: a prospective study of 81 cases. Acta Orthop Scand.

[14] Pradhan A, Aboelmagd T, Richardson L, Olivarius-McAllister J, Ralhan S, Deakin M (2022). Outcomes for non-operatively managed fracture neck of femur patients: a single-institution study. Injury.

[15] Omura T, Ito H, Araki A (2021). Which is a better predictor for adverse events in older adults with diabetes, frailty or higher-level functional incapacity?. Geriatr Gerontol Int.

[16] Haleem S, Heinert G, Parker MJ (2008). Pressure sores and hip fractures. Injury.

